# Exploring the attitudes and experiences of Hungarian primary care physicians on the utilisation of digital health solutions

**DOI:** 10.1186/s12875-024-02642-8

**Published:** 2024-11-14

**Authors:** Edmond Girasek, Bence Döbrössy, Julianna Boros, Zsuzsa Győrffy

**Affiliations:** 1 Faculty of Medicine, Institute of Behavioural Sciences, Semmelweis University, Budapest, Hungary; 2 Hungarian Demographic Research Institute, Budapest, Hungary

**Keywords:** Digital health, Primary care, Family practice, Digital technologies, Telemedicine, Attitudes towards digital health

## Abstract

**Background:**

During the COVID-19 pandemic, digital health solutions ensured the continuity of care especially in primary healthcare practices. COVID-19 accelerated the adoption of digital health solutions. The aim of this study is to describe the digital health-related attitudes and experiences of Hungarian Primary Care Physicians (PCPs) and also analyse the socio-demographic effects on digital health attitudes among PCPs.

**Methods:**

This study used a quantitative and qualitative mixed methodological approach to examine the digital health-related attitudes and experience of Hungarian PCPs. As part of the "E-physicians and E-patients in Hungary" survey, we conducted an online survey among medical doctors working in Hungary between July 2021 to May 2022. A total of 1,774 questionnaires were received, consisting of 1,576 medical doctors and 198 dentists. Among the medical doctors there were 415 primary care physicians (PCPs). In addition to the online questionnaire survey, qualitative research in the form of semi-structured interviews with doctors was also conducted. These interviews took place between October 2021 and June 2022. A total of 62 interviews were conducted,19 with PCPs.

**Results:**

Primary care physicians are more open to technologies that facilitate communication and collaboration with patients, while showing less interest in technologies that support clinical work. Of the demographic variables (age, gender, type of settlement), age was found to have the most significant effect on digital health knowledge, use and intended use. Both the interviews and the multivariate analysis indicate that individuals with greater knowledge, expertise, and experience are more likely to perceive the advantages of digital solutions. This highlights the importance of training, especially given the significant aging population among Hungarian primary care physicians, who may not be accustomed to using these tools naturally. The way PCPs perceive patient expectations regarding the use of digital health tools has a significant impact on the PCPs’ use and intended use of digital tools. When looking at perceived patient needs among PCPs the effect of age and municipality type of PCPs is significant. As age increases, the perception of patient needs decreases (from 5.02 to 4.47), and by municipality type, the average number of perceived needs decreases as one moves from larger cities to smaller municipalities (from 4.85 to 4.14).

**Conclusions:**

Digital health solutions have the potential to enhance the work of PCPs, but successful implementation requires addressing specific needs, demographic differences, and challenges faced by PCPs. Development of infrastructure, education, and institutional support is necessary to ensure more efficient and higher-quality healthcare delivery through the use of digital technologies.

**Supplementary Information:**

The online version contains supplementary material available at 10.1186/s12875-024-02642-8.

## Background

Primary care is a complex form of health care provision. It is defined by the WHO as “the first level of contact for the population with the healthcare system, bringing healthcare as close as possible to where people live and work. It addresses the main health problems in the community, providing preventive, curative and rehabilitative services. Primary care goes beyond services provided by primary care physicians to encompass other health professionals such as nurses, pharmacists, auxiliaries, and community health workers. Primary care as the first point of care, where primary care providers deliver people-centred care, has the potential to respond to major health challenges and to promote health for all” [1].

Digital health includes information and communication technology use for health, mobile technology use for health and telehealth, i.e. the remote provision of care and monitoring for patients using digital tools [2].

The literature identified 5 major uses of digital health in primary care practices [3]: first, to support services like teleconsultations, diagnosis, and testing; second, to inform and educate patients; third, to monitor patients; fourth, e-prescriptions; fifth, enhance system decision-making.

Before 2020 digital technology acceptance and implementation in primary care have been reluctant and delayed [4]. COVID-19 kicked in the door. In April 2020, during the first wave of the pandemic, 64–80% of Primary Care Physicians (PCP) in the United Kingdom and the Netherlands used video consultations, 13% used telemonitoring, and 73% used electronic asynchronous consultations [4]. By April 2020 telephone consultations in US outpatient centres accounted for 65.4% of primary care consultations [5]

According to the OECD report’Health at a Glance 2021’ in 2021, 93% of Primary Care Practices used Electronic Medical Records (EMR) in the 24 OECD countries [6]. There was great variation. While 100% of PCPs used EMR in 15 countries (including Hungary), only 42% in Japan, 40% in Switzerland, and 30% in Mexico and Poland. Countries resisting EMR use in primary care, like Japan and Switzerland, may seem to have less to do with financial resources than with professional culture.

According to a 2023 WHO report, 84% of WHO European Region member states use Electronic Health Records (EHR) regularly in primary care. In secondary care this number is 78% and in tertiary care it is only 69% [7].

The 2022 Commonwealth Fund International Health Policy Survey of PCP [8] found that two years into the pandemic, the majority of PCPs in half of the countries surveyed reported seeing more than a quarter of their patients through telehealth. In the United Kingdom, more than a quarter of physicians saw most of their patients via telehealth. The majority of PCPs reported that implementing a telehealth platform was “somewhat” or “very” easy. But only three of 10 German physicians and four of 10 Swiss physicians reported the same.

In Hungary, there are significant regional disparities in terms of the number of Primary Care Physicians (PCPs) [9, 10]. The age distribution of PCPs indicates a significant aging trend: 55,9% of the practicing PCPs are 60 years old or older [11]. During COVID-19 digital health solutions ensured the continuity of care in primary care provision. Therefore, it is of utmost importance to incorporate digital health technologies into primary care, as they have the potential to alleviate the burden on PCPs. By leveraging digital health solutions, the workload of PCPs can be significantly reduced, leading to more efficient and accessible healthcare services. However, it is crucial to consider the differing needs between age groups when implementing digital health technologies, to ensure that these solutions effectively support all practitioners. A study by Tajirian et al. supports the notion that EHR use may contribute to the burnout of medical doctors. Combating physician burnout by limiting unneeded administrative burdens of EHRs through efficient implementation of systems is important [12]. If younger physicians acted like older physicians in EHR use, there would be 37,600 extra patient visits per week in the U.S., workforce. [13].

In the European ranking of the Digital Economy and Society Index (DESI) [14], Hungary was placed 22nd in 2022, with significantly lower scores in the Human Capital and Integration of Digital Technologies sub-indicators compared to the European average.

Hungary had no national digital health strategy until 2021. The legal environment for digital healthcare was fragmented and incomplete. Although the technology needed was present, having one of the best broadband coverage according DESI [14], the legislation on how it may be used, what can be financed and what measures may be taken was lacking [15]. Hence not a lot of doctors took the risk of engaging in this unregulated activity. That quickly changed as a result of the pandemic. As soon as the lockdown created the need for digital health the government issued 7 governmental and ministerial decrees regulating the use and financing of digital health. Not only did they make ePrescriptions, telemedicine and teleconsultations easier and safer to use, they also provided the framework of how doctors can be reimbursed for using them, which is a great incentive [16].

In this paper, we examine the changes related to digital health cultural shifts in terms of usage and attitudes of Hungarian PCP. Digital health solutions could potentially combat the issues caused by mentioned regional physician shortages and improve access to health care. However, this depends on the willingness of practitioners to use it, which highlights the value of our study.

## Research

The aim of the study is to describe the digital health-related attitudes and experiences of Hungarian PCPs and compare them to non-primary care medical doctors while also analysing the socio-demographic effects on digital health attitudes among PCPs.

Our research questions are:

- What are the digital health-related attitudes and experiences of Hungarian PCPs?
- Are there any differences between the digital health-related attitudes and experiences of PCPs and non-primary care medical doctors in Hungary and if so, what are they?
- Are there any effects of gender, age and settlement type on digital health-related attitudes and experiences of Hungarian PCPs?

This study used a mixed methodological approach to answer the stated research questions. A quantitative survey and semi-structured interview research were conducted between autumn 2021 and spring 2022.

The study “E-physicians and E-patients in Hungary” was conducted by the Digital Health Working Group of the Institute of Behavioural Sciences at Semmelweis University as part of the OTKA-FK 134372 research program.

### Quantitative study

As part of the “E-physicians and E-patients in Hungary” study, we conducted an online survey among medical doctors working in Hungary. The questionnaire was made available online in a self-administered format from July 2021 to May 2022. Developed internally by our research team [17]. Respondents were contacted through a newsletter sent by the Hungarian Medical Chamber (HMC) and received personalised email invitations. Data collection was significantly complicated by the third and fourth waves of the COVID-19 pandemic, which affected all doctors involved in patient care. Consequently, the survey was conducted in multiple waves. A newsletter was sent to HMC members in autumn 2021, followed by a targeted email survey with physicians in agreement with the HMC in spring 2022, after the COVID-19 epidemic had subsided.

A total of 1774 questionnaires were received, consisting of 1576 medical doctors and 198 dentists. Among the medical doctors there are 415 PCPs. Given that there were approximately 4,473 PCPs practicing in Hungary in 2021 [24], the response rate is more than 9% of the total PCP population [18]. The age distribution of respondents is slightly younger than the age distribution of doctors working in primary care in Hungary. Proportionately more younger (6.3% vs 1.4%), more middle-aged (67.1% vs 60.96) and fewer older respondents (26.6% vs 37.64%) took our survey than would be expected from the number of doctors working in primary care in Hungary.

The questionnaire covers 4 main areas (socio-demographic data, information related to medical work, digital technologies, attitudes towards digital health) and contains 48 questions in total. The questions were arranged in blocks by topic. Only data on questions related on digital health devices using experiences and attitudes were included in the analysis of this article. The questionnaire can be found in Annex 1.

Correction weighting was applied to the responses based on statistics obtained from the National Register of Medical Doctors, which were provided by the Directorate of Human Resources Development of the Ministry of Health. The correction weighting considered factors such as gender, age, and county of workplace. This correction was necessary due to slight variations in the sample compared to the main distributions of the Register. The weight variable had a mean value of 1, a first quartile value of 0.6255, and a third quartile value of 1.1942.

Data analysis was performed using IBM Statistics (SPSS 28) [19]. The statistical data processing involved examining distributions, conducting cross-tabulation analyses, and performing chi-square tests. Cross-tabulation analyses were conducted using the chi-square test, means were compared using analysis of variance (ANOVA), and linear regression models were also utilised. The following tables are bolded where *p* < 0.05.

The variables used in the analysis were derived from the raw data by aggregating the responses to each question on an individual basis (e.g., assessing the perceived benefits of digital health solutions). For the variables on advantages, disadvantages and patient needs, the marked responses were summed up, so the resulting variables shows how many of these were marked. For the variable on what would be necessary to adopt digital healthcare solution, two separate variables were extracted; one related to training, and knowledge transfer (Postgraduate training, other continuing education, available professional materials, professional protocols, data security protocols) while the other counted the remaining factors (financial incentives, availability of technology, accessibility, peer recommendation, evidence-based practice, ethics legislation, time available from work, patient engagement, greater collaboration). Postgraduate training is vital for the adaption of digital solutions. Enhancing relevant skillsets and knowledge through lifelong learning and continuous professional development is a vital prerequisite in this field as this type of training has only very recently became part of the medical curriculum. That is why we considered it as a variable on its own, and it helps us to detect sociodemographic differences among PCPs in access to knowledge. The other items have been considered as one variable because, in addition to knowledge, they have been summarised as systemic tools that cannot be created at the PCP level, so they have not been created as multiple variables. The mean values of these summed-up variables were examined in to identify the socio-demographic patterns. The mean values and comparison between Primary Care Physicians and Non-Primary Care Physicians can be seen in Table 1.

**Table 1:** The main indicators of computed aggregated variables

	Non-Primary Care Pysicians					Primary Care Physician (PCP)					Total				
	Mean	N	Std. Deviation	Minimum	Maximum	Mean	N	Std. Deviation	Minimum	Maximum	Mean	N	Std. Deviation	Minimum	Maximum
**How many digital health solutions are known by you? (*p* < 0.001)**	**9,21**	**1160**	**3,05**	**0,00**	**13,00**	**8,53**	**415**	**3,07**	**0,00**	**13,00**	**9,03**	**1575**	**3,07**	**0,00**	**13,00**
How many technologies do you use frequently or daily? (*p* = 0.077)	2,25	1160	1,83	0,00	11,00	2,44	415	1,98	0,00	12,00	2,30	1575	1,87	0,00	12,00
**How much technology would you like to use in the future? (*p* < 0.001)**	**5,02**	**1160**	**3,41**	**0,00**	**13,00**	**4,05**	**415**	**3,18**	**0,00**	**13,00**	**4,76**	**1575**	**3,38**	**0,00**	**13,00**
How many advantages do digital healthcare solutions have? (*p* = 0.458)	7,76	1160	4,00	0,00	17,00	7,93	415	4,06	0,00	17,00	7,80	1575	4,02	0,00	17,00
How many disadvantages do digital healthcare solutions have? (*p* = 0.770)	4,23	1160	2,08	0,00	11,00	4,26	415	2,11	0,00	11,00	4,24	1575	2,09	0,00	11,00
**How many solutions do your patients express a need for? (*p* < 0.001)**	**3,66**	**1160**	**1,99**	**0,00**	**8,00**	**4,47**	**415**	**1,91**	**0,00**	**8,00**	**3,87**	**1575**	**2,00**	**0,00**	**8,00**
How many factor would be necessary (training, protocols, knowledge sharing) for you to adopt digital healthcare solutions? (*p* = 0.261)	2,50	1160	1,50	0,00	5,00	2,60	415	1,42	0,00	5,00	2,53	1575	1,48	0,00	5,00
Apart from training or knowledge sharing, how many factor would be needed for you to use digital healthcare solutions? (*p* = 0.986)	3,60	1160	1,77	0,00	7,00	3,60	415	1,62	0,00	7,00	3,60	1575	1,73	0,00	7,00

#### Qualitative research

In addition to the online survey, qualitative research in the form of semi-structured interviews was conducted with doctors between October 2021 and June 2022. A total of 62 interviews were conducted, 19 with PCPs. The age range of the participating PCPs was 25 to 64 years (average: 47,3 years), with 11 women and 8 men. Among them, 6 practiced in Budapest, 9 in rural towns, and 4 in villages.

Purposive sampling was based on the following criteria: (1) physicians who are actively involved in patient care, (2) work in Hungary and (3) have experience in digital health solutions. The “information power” criteria were based on (a) the aim of the study, (b) sample specificity, (c) the use of established theory, (d) quality of dialogue, and (e) analysis strategy [20]. Since the aim of our study was to assess different aspects of the digital transformation in the medical profession (see Supplementary Material 2 for interview guide) and the analytical framework was thematic analysis, a larger sample size was chosen. However, for the presented analysis only interviews with primary care physicians were included [21].

The interview guide, developed from the study aims and literature review, was implemented in Hungarian by trained interviewers. Before full implementation, they conducted pilot interviews on a physician sample (*n* = 4) and modifications were made based on the feedback. The following topics were included: work and career choice, technological changes in medical field over the past decades, the different types of digital health devices and services they use/know, how the doctor-patient relationship changed since the start of their career, what they think about the future role of digital health (See Annex 2 for the complete interview guide).

Interviews were audio recorded in person and online (Zoom video call), with an average interview length of 60 min. All audio recorded interviews were transcribed verbatim and each transcript was anonymised and attributed a unique code. The interviewers checked the transcriptions for accuracy. Then, the final texts were sent back to each interviewee for confirmation. An inductive thematic approach was used to analyse the data and identify patterns of themes. Five researchers discussed and developed all themes and subthemes and clarified any discrepancies during the coding. They then developed the final thematic map, which was determined in mutual agreement. The results are supported by anonymised quotes from different participants. All interviews were coded using Atlas.ti 6.0. software [22].

The research has an ethical approval by TUKEB (Hungarian Scientific Research and Research Ethics Committee) with the reference number IV-10927–1 TUKEB.

## Results

### Demographic profile

In our medical survey, a total of 1,575 doctors responded to the questionnaire, including 415 PCPs. In the following analysis, we examine their responses and compare them with those of the other participating doctors (Table 2). Notably, there is a significant difference in age between responding PCPs and other doctors. PCPs show a higher representation in the 65 years or older category (26.6% vs 19.0%) and in the 36–64 age group (67.1% vs 48.8%), which is consistent with the overall medical population, as PCPs tend to have higher average ages. Furthermore, there is a significant difference in the workplace locality, which can be explained by the nature of the work. Non-PCP doctors are more likely to work in institutions located in larger cities.

**Table 2:** Demographic profile of the respondents to the quantitative (survey) study

			Non-PCPs	PCPs
Gender (*p* > 0.05)	male	n	500	158
		%	43.1%	38.1%
	female	n	660	257
		%	56.9%	61.9%
	total		1160	415
**Settlement type of workplace (*p* < 0.05)**	**Capital**	**n**	**421**	**84**
		**%**	**36.4%**	**20.3%**
	**County seat**	**n**	**442**	**91**
		**%**	**38.2%**	**22.0%**
	**Town**	**n**	**278**	**159**
		**%**	**24.0%**	**38.5%**
	**Village, countryside**	**n**	**16**	**79**
		**%**	**1.4%**	**19.1%**
	**total**		**1157**	**413**
**Age group (*p* < 0.05)**	**35 years old at maximum**	**n**	**373**	**26**
		**%**	**32.1%**	**6.3%**
	**36–64 years old**	**n**	**567**	**278**
		**%**	**48.8%**	**67.1%**
	**65 years old or older**	**n**	**221**	**110**
		**%**	**19.0%**	**26.6%**
	**total**		**1161**	**414**

The text and values are bolded where *p* < 0.05

### Knowledge of technologies

In the research, we inquired about the respondents’ familiarity with various digital healthcare technologies. Table 3 shows that PCPs are less familiar with international literature tracking online, augmented reality, virtual reality, 3D printing, artificial intelligence applications in healthcare, robotics, and nanotechnology. On the other hand, fewer non-PCPs are familiar with telemedicine (remote consultations) and social media for patient communication, which facilitate easier communication with patients.

**Table 3:** Knowledge of digital health technologies – Do you know these technologies?

		Non-PCP medical doctors (*n* = 1160)	PCPs (*n* = 415)
Participating in online conferences and trainings	n	1130	401
	%	97.9%	96.6%
**Tracking international literature, trends, and data online**	**n**	**1078**	**337**
	**%**	**93.2%**	**82.0%**
**Telemedicine, remote visit**	**n**	**969**	**385**
	**%**	**84.0%**	**92.8%**
Smartphone applications, apps	n	1007	352
	%	87.6%	85.2%
**Healthcare-related social media, communication with patients, information sharing**	**n**	**851**	**341**
	**%**	**73.7%**	**82.2%**
Home-usable healthcare sensors, smart devices	n	1004	362
	%	86.9%	87.7%
Portable diagnostic devices (e.g., ultrasound, mobile ECG)	n	993	364
	%	86.3%	88.1%
**Augmented reality (e.g., surgical practice)**	**n**	**483**	**103**
	**%**	**41.9%**	**24.8%**
**Use of Virtual Reality (e.g., pain management, psychotherapy)**	**n**	**426**	**115**
	**%**	**36.9%**	**27.9%**
**3D printing (e.g., dental, surgical solutions)**	**n**	**678**	**189**
	**%**	**59.0%**	**45.7%**
**Artificial intelligence solutions in medical decision-making (radiology, pathology, ophthalmology, diagnostic solutions)**	**n**	**647**	**176**
	**%**	**56.0%**	**42.5%**
**Robotics (e.g., surgical robots, disinfection robots, delivery robots)**	**n**	**628**	**165**
	**%**	**54.5%**	**39.8%**
**Nanotechnology (e.g., ingestible diagnostic devices)**	**n**	**791**	**252**
	**%**	**68.7%**	**61.0%**

The text and values are bolded where *p* < 0.05

Among primary care physicians, the socio-demographic correlations for the aggregated variables were investigated (Table 4). We find that gender and type of settlement have no effect, but that the correlation with age is significant: the number of known digital health tools decreases with age, with a mean of 10.17 for the youngest age group and 7.53 for the over-65 s.

**Table 4:** How many digital health solutions are known by you? – Among PCP respondents

How many digital health solutions are known by you?				
		Mean	N	Std. Deviation
Gender (*p* > 0.171)	Male	8.41	158	3.36
	Female	8.61	257	2.88
	Total	8.53	415	3.07
**Age group (*p* < 0.001)**	**35 years old at maximum**	**10.17**	**26**	**2.68**
	**36–64 years old**	**8.77**	**278**	**2.92**
	**65 years old or older**	**7.53**	**110**	**3.24**
	**Total**	**8.53**	**415**	**3.07**
Settlement type of workplace (*p* = 0.519)	Capital	8.81	84	2.99
	County seat	8.76	91	3.23
	Town	8.28	159	2.95
	Village, country	8.46	79	3.22
	Total	8.53	413	3.07

The text and values are bolded where *p* < 0.05

### The use of technologies

The knowledge of digital healthcare technologies is closely related to their usage. The following table (Table 5) illustrates the frequency of usage for different technologies. PCPs utilise telemedicine social media for patient communication, and portable diagnostic devices significantly more frequently than non-PCPs.

**Table 5:** Use of digital health technologies – Do you use these technologies frequently or daily?

		Non-PCPs (*n* = 1160)	PCPs (*n* = 415)
Participating in online conferences and trainings	n	410	156
	%	35.4%	37.5%
**Tracking international literature, trends, and data online**	**n**	**597**	**124**
	**%**	**51.7%**	**30.0%**
**Telemedicine, remote visit**	**n**	**315**	**260**
	**%**	**27.4%**	**62.8%**
**Smartphone applications, apps**	**n**	**540**	**154**
	**%**	**46.8%**	**37.2%**
**Healthcare-related social media, communication with patients, information sharing**	**n**	**161**	**120**
	**%**	**13.9%**	**29.0%**
Home-usable healthcare sensors, smart devices	n	273	85
	%	23.6%	20.5%
**Portable diagnostic devices (e.g., ultrasound, mobile ECG)**	**n**	**165**	**91**
	**%**	**14.3%**	**22.0%**
**Augmented reality (e.g., surgical practice)**	**n**	**17**	**4**
	**%**	**1.5%**	**1.0%**
**Use of Virtual Reality (e.g., pain management, psychotherapy)**	**n**	**34**	**9**
	**%**	**2.9%**	**2.2%**
**3D printing (e.g., dental, surgical solutions)**	**n**	**5**	**3**
	**%**	**0.4%**	**0.7%**
**Artificial intelligence solutions in medical decision-making (radiology, pathology, ophthalmology, diagnostic solutions)**	**n**	**70**	**4**
	**%**	**6.1%**	**1.0%**
**Robotics (e.g., surgical robots, disinfection robots, delivery robots)**	**n**	**12**	**1**
	**%**	**1.0%**	**0.2%**
**Nanotechnology (e.g., ingestible diagnostic devices)**	**n**	**11**	**1**
	**%**	**1.0%**	**0.2%**

The text and values are bolded where *p* < 0.05

However, fewer PCP use online platforms and smartphone applications for international literature tracking. There is a significant difference in several technologies, although the sample size is extremely low. Of course, these differences are expected due to different work tasks, methods and tools compared to other medical fields.

In terms of the use of technology, it can be observed that among primary care physicians, the effect of gender and age is noticeable (Table 6). Women use significantly more technology, and again for age, younger people use more devices than older people (4.11 and 1.68 on average for the young and old age groups respectively).

**Table 6:** How many technologies do you use frequently or daily? – Among PCP respondents

How many technologies do you use frequently or daily?				
		Mean	N	Std. Deviation
**Gender (*p* > 0.012)**	**Male**	**2.13**	**158**	**1.87**
	**Female**	**2.63**	**257**	**2.03**
	**Total**	**2.44**	**415**	**1.98**
**Age group (*p* < 0.001)**	**35 years old at maximum**	**4.11**	**26**	**2.19**
	**36–64 years old**	**2.58**	**278**	**1.94**
	**65 years old or older**	**1.68**	**110**	**1.71**
	**Total**	**2.44**	**415**	**1.98**
Settlement type of workplace (*p* = 0.059)	Capital	2.50	84	1.86
	County seat	2.62	91	1.91
	Town	2.13	159	1.83
	Village, country	2.81	79	2.40
	Total	2.44	413	1.98

The text and values are bolded where *p* < 0.05

### Attitudes towards the use of digital devices

In the survey, we measured attitudes related to digital device usage employing variables derived from the following questionnaire questions by counting the occurrences of items in the responses:

- How many digital health solutions are known by you?
- How many technologies do you use frequently or daily?
- How much technology would you like to use in the future?
- How many advantages do digital healthcare solutions have?
- How many disadvantages do digital healthcare solutions have?
- How many digital health solutions do your patients express a need for?
- How much support would be necessary (training, protocols, knowledge sharing) for you to adopt digital healthcare solutions?
- Apart from training or knowledge sharing, how much support would be needed for you to use digital healthcare solutions?

There is a significant difference between PCPs and non-PCPs in terms of patient demands and knowledge of technologies (Table 1). PCPs experienced an average of 4.47 digital technology-related demands from their patients, while the number for other physicians was 3.66. On average, PCPs are familiar with fewer technologies, with a score of 8.53, compared to 9.21 for PCPs. No significant differences were observed in other variables.

### Perceived advantages

We examined the perceived benefits in detail among PCPs and non-PCP medical doctors (Table 7). Based on these results, more PCPs believe that the use of digital tools improves diagnostic capabilities, reduces the number of doctor-patient meetings, and makes healthcare delivery more efficient. In contrast, fewer PCPs than other physicians believe that the use of digital tools is convenient, saves time for the doctor, and reduces the likelihood of errors.

**Table 7:** Perceived advantages of digital health solutions

		Non-PCPs (*n* = 1160)	PCPs (*n* = 415)
Improved efficiency	n	806	284
	%	69.5%	68.6%
Enhanced safety	n	333	112
	%	28.7%	27.0%
**Improved diagnostic capabilities**	**n**	**493**	**200**
	**%**	**42.5%**	**48.2%**
Reduced burnout	n	337	106
	%	29.1%	25.5%
Increased patient adherence and collaboration	n	625	236
	%	53.9%	56.9%
**Convenient**	**n**	**820**	**262**
	**%**	**70.6%**	**63.1%**
**Reduced the number of in-person doctor-patient meetings**	**n**	**645**	**289**
	**%**	**55.6%**	**69.6%**
**Save time for the doctor**	**n**	**680**	**217**
	**%**	**58.6%**	**52.3%**
Save time for the patient	n	736	278
	%	63.4%	67.0%
Enable faster access to healthcare	n	582	225
	%	50.2%	54.2%
**Make your work more efficient**	**n**	**544**	**236**
	**%**	**46.9%**	**56.9%**
Engage patients more actively in their own healing process	n	529	207
	%	45.6%	49.9%
Improve the quality of care	n	427	174
	%	36.8%	41.9%
**Reduce the likelihood of errors**	**n**	**245**	**62**
	**%**	**21.1%**	**15.0%**
Generate additional income for doctors	n	100	29
	%	8.6%	7.0%
Increase patient satisfaction	n	576	191
	%	49.6%	46.0%
Improve doctor-patient communication	n	522	182
	%	45.0%	43.9%

The text and values are bolded where *p* < 0.05

In terms of benefits, among PCPs, only age has a significant effect, with younger age groups also seeing more advantages from digital health solutions (Table 8).

**Table 8:** How many advantages do digital healthcare solutions have? – Among PCP respondents

How many advantages do digital healthcare solutions have?				
		Mean	N	Std. Deviation
Gender (*p* = 0.210)	Male	8.25	158	4.17
	Female	7.73	257	3.99
	Total	7.93	415	4.06
**Age group (*p* < 0.001)**	**35 years old at maximum**	**10.27**	**26**	**3.65**
	**36–64 years old**	**8.14**	**278**	**3.92**
	**65 years old or older**	**6.83**	**110**	**4.21**
	**Total**	**7.93**	**415**	**4.06**
Settlement type of workplace (*p* = 0.615)	Capital	7.98	84	4.00
	County seat	8.27	91	3.75
	Town	7.62	159	4.08
	Village, country	8.14	79	4.47
	Total	7.94	413	4.06

The text and values are bolded where *p* < 0.05

In the qualitative study, all PCPs observed numerous benefits of digital healthcare. Most of them emphasised that it speeds up administrative tasks, and that telemedicine makes patient care more efficient.

“We can handle much more patient care this way, it is much better documented, which is increasingly necessary, and I think the quality of care also improves because it can be traced back.” (Demo_01, 49-year-old female).

Additionally, it is seen as an advantage that online access to electronic medical records facilitates patient information, and doctors can easily review medical histories.

“The patient can look at their own test results, there is no need for printing, which is a significant cost-saving on a national scale. It also facilitates telemedicine; the patient doesn't have to run around for their test results. When I call a patient, I always read through what the Electronic Health Records (EESZT) say, which makes it much simpler, especially because the patient is not sitting right next to me.” (Demo_33, 25-year-old male).

From a professional perspective, it also eases and speeds up consultations with specialists, thus eliminating geographical disadvantages:

“In many places, there is a CT scan or CT device, and we take the image, but, for example, I worked in Ózd, and we did the CT scan there, but the actual analysis of the CT scan happened online in Miskolc. If something happened, like a brain haemorrhage, then the local doctor immediately contacted the neurosurgeon, the neurosurgeon looked at it, and I already received the results, and then we sent the patient immediately for surgery to the neurosurgery department.” (Demo_62, 64-year-old male).

### Perceived disadvantages

Similar to the perceived benefits, we also examined perceived disadvantages (Table 9). It is evident that more PCPs believe that the use of digital tools frustrates patients, increases the administrative burden on doctors, poses extra costs to practices, and increases the risk of burnout. In contrast to non-PCPs, fewer PCPs think that it can lead to overdiagnosis, that patients misinterpret the shared health data, and that faulty technology jeopardises patients' recovery.

**Table 9:** Perceived disadvantages of digital health solutions

		Non-PCPs (*n* = 1160)	PCPs (*n* = 415)
Decreased quality of care	n	365	114
	%	31.5%	27.5%
**Frustration among patients**	**n**	**216**	**109**
	**%**	**18.6%**	**26.3%**
**Potential for overdiagnosis**	**n**	**471**	**111**
	**%**	**40.6%**	**26.7%**
**Misinterpretation of shared health data by patients**	**n**	**784**	**246**
	**%**	**67.6%**	**59.3%**
Increased possibility of misunderstandings in doctor-patient communication	n	583	201
	%	50.2%	48.4%
**Faulty technology jeopardising patient recovery**	**n**	**315**	**86**
	**%**	**27.2%**	**20.7%**
Compromised confidentiality of patient data	n	512	162
	%	44.1%	39.1%
**Increased administrative burdens on doctors**	**n**	**574**	**249**
	**%**	**49.5%**	**60.0%**
**Additional costs for practices**	**n**	**365**	**189**
	**%**	**31.5%**	**45.5%**
Limited patient proficiency in using digital technologies, placing a burden on the treating physician	n	612	238
	%	52.8%	57.5%
**Increased likelihood of burnout**	**n**	**110**	**64**
	**%**	**9.5%**	**15.4%**

The text and values are bolded where *p* < 0.05

In terms of perceived disadvantages among primary care physicians (Table 10), only a gender displayed a significant correlation, with women reporting more disadvantages of digital health solutions than men (4.57 vs. 4.08).

**Table 10:** How many disadvantages do digital healthcare solutions have? – Among PCP respondents

How many disadvantages do digital healthcare solutions have?				
		Mean	N	Std. Deviation
**Gender (*p* = 0.019)**	**Male**	**4.57**	**158**	**2.28**
	**Female**	**4.08**	**257**	**1.98**
	**Total**	**4.26**	**415**	**2.11**
Age group (*p* = 0.287)	35 years old at maximum	4.89	26	2.49
	36–64 years old	4.21	278	2.09
	65 years old or older	4.26	110	2.07
	Total	4.26	415	2.11
Settlement type of workplace (*p* = 0.261)	Capital	4.55	84	2.19
	County seat	4.43	91	2.30
	Town	4.17	159	2.08
	Village, country	3.97	79	1.86
	Total	4.27	413	2.11

The text and values are bolded where *p* < 0.05

The disadvantages of digital healthcare were also discussed during the interviews. The depersonalisation of the doctor-patient relationship, is one of the most important issues as personal encounters with patients are often missed due to telemedicine.

“The technology cannot replace the personal encounter, and maybe it is not meant to. That became quite clear. So, the goal is not to have this robotic type of appointment, at least I hope not in the future. It has been proven that the doctor-patient relationship is primarily personal, and it should remain that way.” (Demo 39, 61-year-old male).

Potential technical problems can also cause difficulties:

“If there is a power outage or no internet connection, everything collapses, and then we are simply tied up, helpless. This is one of the drawbacks, that everything collapses and the world stops.” (Demo 62, 64-year-old male).

Many also highlighted that it becomes more difficult to separate work and personal life, to set boundaries, resulting in a significant increase in doctors' workload and burnout.

“After the office hours, I don't want to deal with it anymore because, you know, it really intensified burnout.” (Demo_34, 46-year-old female).

“It happens to me too that the browser keeps the email window of the practice in the district open, and sometimes unintentionally, even on weekends, I find myself looking at it, although I shouldn't have anything to do with it.” (Demo_41, 34-year-old male).

These quotes reflect the concerns and challenges expressed by the participants regarding the depersonalization of the doctor-patient relationship, technical issues, and the impact on work-life balance.

### Perceived patient needs regarding digital health

Regarding demands expressed by patients, there is a significant difference between PCPs and non-PCPs (Table 11). With two exceptions, in all cases of patient demands, it is evident that more PCPs reported these needs than non-PCPs. These needs include email communication, sharing and discussing images and test results, teleconsultation, the ability for patients to track their health status on smartphone, home health sensor use, and the use of social media for communication with the doctor.

**Table 11:** Perceived patient needs regarding digital health

		Non-PCPs (*n* = 1160)	PCPs (*n* = 415)
**Email communication**	**n**	**879**	**394**
	**%**	**75.7%**	**94.9%**
Scheduling appointments online	n	729	278
	%	62.8%	67.0%
**Sharing and discussing images and test results**	**n**	**820**	**360**
	**%**	**70.7%**	**86.7%**
**Teleconsultation (via Skype or video chat)**	**n**	**531**	**228**
	**%**	**45.8%**	**54.9%**
**Monitoring changes in their health status through their smartphones**	**n**	**246**	**117**
	**%**	**21.2%**	**28.2%**
**Using home health sensors**	**n**	**195**	**98**
	**%**	**16.8%**	**23.6%**
Recommending websites with valid medical information	n	453	183
	%	39.1%	44.1%
**Using social media for communication with you**	**n**	**388**	**195**
	**%**	**33.4%**	**47.0%**

The text and values are bolded where *p* < 0.05

When looking at the perceived patient needs among PCPs (Table 12), the effect of age and municipality type is significant. As age of PCPs increases, the perception of patient needs decreases (from 5.02 to 4.47), and by municipality type, the average number of perceived needs decreases as one moves from larger cities to smaller municipalities (from 4.85 to 4.14).

**Table 12:** How many needs do you perceive from patients for digital health solutions?? – Among PCP respondents

How many needs do you perceive from patients for digital health solutions?				
		Mean	N	Std. Deviation
Gender (*p* = 0.470)	Male	4.38	158	1.96
	Female	4.52	257	1.88
	Total	4.47	415	1.91
**Age group (*p* < 0.001)**	**35 years old at maximum**	**5.02**	**26**	**2.04**
	**36–64 years old**	**4.69**	**278**	**1.89**
	**65 years old or older**	**3.77**	**110**	**1.74**
	**Total**	**4.47**	**415**	**1.91**
**Settlement type of workplace (*p* = 0.043)**	**Capital**	**4.85**	**84**	**1.91**
	**County seat**	**4.68**	**91**	**1.78**
	**Town**	**4.31**	**159**	**1.82**
	**Village, country**	**4.14**	**79**	**2.13**
	**Total**	**4.47**	**413**	**1.91**

The text and values are bolded where *p* < 0.05

### What is needed to use digital health technologies?

More PCPs than non-PCPs responded that they would need financial incentives, postgraduate training, other continuing education opportunities, and better cooperation and commitment from patients (Table 13). In contrast, fewer PCPs considered the accessibility of technologies and evidence-based research to be necessary.

**Table 13:** What is needed to use digital health technologies?

		Non-PCPs (*n* = 1160)	PCPs (*n* = 415)
**Financial incentives (e.g., support for acquiring certain tools)**	**n**	**759**	**317**
	**%**	**65.4%**	**76.4%**
**Postgraduate training**	**n**	**386**	**165**
	**%**	**33.3%**	**39.8%**
**Other training opportunities**	**n**	**483**	**206**
	**%**	**41.6%**	**49.6%**
Accessible professional materials (documents, online training, etc.)	n	711	241
	%	61.3%	58.2%
**Availability and accessibility of technologies**	**n**	**803**	**264**
	**%**	**69.2%**	**63.6%**
Recommendations from colleagues	n	185	52
	%	15.9%	12.5%
**Evidence-based research**	**n**	**493**	**139**
	**%**	**42.5%**	**33.5%**
Ethical and legal regulations	n	669	220
	%	57.7%	53.0%
Professional protocols	n	726	269
	%	62.6%	65.0%
Data security protocols	n	599	197
	%	51.6%	47.5%
Dedicated time within working hours	n	765	294
	%	65.9%	70.8%
**Patient commitment and increased collaboration**	**n**	**502**	**207**
	**%**	**43.2%**	**49.9%**

The text and values are bolded where *p* < 0.05

As mentioned above, the answers to the question "what would be needed" have been used to create two separate variables, one for training, knowledge and the other for everything else (e.g. financial support, infrastructure). Looking at the means of the new variables (Tables 14 and 15), it is noticeable that women have a significantly higher mean for knowledge needs (2.72) than men (2.41).

**Table 14:** How many factors would be necessary (training, protocols, knowledge sharing) for you to adopt digital healthcare solutions? – Among PCP respondents

How many factors would be necessary (training, protocols, knowledge sharing) for you to adopt digital healthcare solutions?				
		Mean	N	Std. Deviation
**Gender (*p* = 0.031)**	**Male**	**2.41**	**158**	**1.46**
	**Female**	**2.72**	**257**	**1.39**
	**Total**	**2.60**	**415**	**1.42**
Age group (*p* = 0.137)	35 years old at maximum	3.12	26	1.27
	36–64 years old	2.59	278	1.44
	65 years old or older	2.51	110	1.39
	Total	2.60	415	1.42
Settlement type of workplace (*p* = 0.586)	Capital	2.54	84	1.33
	County seat	2.78	91	1.53
	Town	2.56	159	1.39
	Village, country	2.54	79	1.45
	Total	2.60	413	1.42

The text and values are bolded where *p* < 0.05

**Table 15:** Apart from training or knowledge sharing, how many factors would be needed for you to use digital healthcare solutions? – Among PCP respondents

Apart from training or knowledge sharing, how many factors would be needed for you to use digital healthcare solutions?				
		Mean	N	Std. Deviation
Gender (*p* = 0.054)	Male	3.41	158	1.55
	Female	3.72	257	1.65
	Total	3.60	415	1.62
**Age group (*p* = 0.024)**	**35 years old at maximum**	**3.61**	**26**	**1.23**
	**36–64 years old**	**3.74**	**278**	**1.61**
	**65 years old or older**	**3.25**	**110**	**1.68**
	**Total**	**3.60**	**415**	**1.62**
Settlement type of workplace (*p* = 0.245)	Capital	3.72	84	1.47
	County seat	3.85	91	1.70
	Town	3.47	159	1.65
	Village, country	3.46	79	1.59
	Total	3.60	413	1.62

The text and values are bolded where *p* < 0.05

### Multivariate model—perceived advantages

In the linear regression analysis, we focus on PCPs, therefore the independent variable is constructed based on a sample of 415 PCPs to build the model.

A linear regression model was performed with the perceived benefits as the dependent variable, using a stepwise variable selection method, incorporating the following explanatory variables:

- How many disadvantages do digital healthcare solutions have?
- How many solutions do your patients express a need for?
- How many digital health solutions are known by you?
- How many technologies do you use frequently or daily?
- What would be necessary (training, protocols, knowledge sharing) for you to adopt digital healthcare solutions?
- Apart from training or knowledge sharing, what else would be needed for you to use digital healthcare solutions?
- Age of respondent
- Settlement type of respondent’s workplace
- Gender of respondent.

The model was run in 6 steps, and the final regression model achieved an R-squared value of 0.350 (Table 16). The strongest impact is shown by the number of frequently or daily used technologies (Beta = 0.286), followed by the demand for knowledge related to digital device usage (0.223), and the perceived needs from patients, which also exert a positive effect on perceived benefits (Beta = 0.166). Additionally, there is a significant correlation with a variable measuring the quantity of infrastructure and other factors associated with digital device usage (Beta = 0.194). Furthermore, gender (Beta = -0.162) also holds significant explanatory power, indicating that males perceive more benefits. There is a negative inverse relationship with perceived disadvantages (Beta = -0.155). Age does not have a significant effect as it is strongly correlated with the usage of digital healthcare devices (Pearsons’ correlation -0.275, *p* < 0.001), therefore it is not separately included in the model.

**Table 16:** Linear regression model, dependent variable is How many advantages do digital healthcare solutions have?

	Unstandardized Coefficients		Standardized Coefficients	t	Sig
	B	Std. Error	Beta		
(Constant)	5,747	0.285		20.132	0.000
How many technologies do you use frequently or on a daily basis?	0.586	0.094	0.286	6.268	0.000
How much support would be necessary (training. protocols. knowledge sharing) for you to adopt digital healthcare solutions?	0.637	0.134	0.223	4.747	0.000
How many solutions do your patients express a need for?	0.353	0.099	0.166	3.558	0.000
Gender of respondent (1 = male; 2 = female)	-1.354	0.340	-0.162	-3.981	0.000
Apart from training or knowledge sharing, how much support would be needed for you to use digital healthcare solutions?	0.487	0.122	0.194	3.976	0.000
How many disadvantages do digital healthcare solutions have?	-0.299	0.081	-0.155	-3.664	0.000

Dependent variable: How many advantages do digital healthcare solutions have?

Analysis of interviews reveals that both the age of responding physicians and the age distribution of their patient population significantly influence perceived patient needs regarding digitization. Age has no significant effect in the linear regression model, but it has a significant background effect on the variables in the model. The interviews demonstrate that the age of PCPs plays a decisive role, as younger doctors are generally more open-minded. However, among older doctors, there are also those who can be well motivated by their younger patients:

“…what the young people teach me, or what I am forced to learn, I can learn it nicely. So I think it's about acquiring these skills.” (Demo_36, 53-year-old female).

A middle-aged PCP (Demo_26) reported that his patients generally have better digital skills and knowledge, which serves as motivation for him:

“I noticed that many people say, 'Finally, the doctor is where everything else is.”

From an age perspective, patients can be divided into two groups: the younger ones are more comfortable navigating the digital world, while the older ones are less so:

“There's no problem with the age group under 50; they are practically well-prepared, except for a few poorer segments. However, there are already problems with the age group over 70. I have a patient who is over 65 and still doesn't have a mobile phone, only a landline, and no internet. This is problematic for them.” (Demo_61, 41-year-old male).

Paediatric PCPs stand out in this regard since their clientele (parents of children) predominantly belongs to the younger generation and therefore is more open to digital technologies.

“Young people […] use these opportunities on a daily basis and are familiar, for example, with sending a photo via email. The less informed ones can use Messenger just fine. As for the older patients, it's not a problem because their children are older, and their children use these devices. So I don't think there is an issue with accessing these things. However, not everyone has a phone to make calls.” (Demo_07, 59-year-old female).

Most interviewees, however, have an optimistic view of the future. They believe that progress is unstoppable, and older people will increasingly become part of it:

“Patients are using health monitoring sensors and diagnostic devices more and more. The more skilled ones record these data through applications and forward them to our email address. […] Different smart devices (e.g., watches) are mainly used by young people, but older individuals can also use them if they are taught. Younger family members can be a great help in this regard. We are moving more and more towards older people using digital devices and being able to monitor themselves.” (Demo_20, 46-year-old female).

## Discussion

During the COVID-19 pandemic and the accompanying restrictions, PCPs who utilised digital healthcare solutions were able to keep on providing health care [23, 24]. Even prior to the COVID-19 outbreak, the use of digital technologies in patient care had significantly increased in several countries, for example the United Kingdom [3]. The pandemic further accentuated this openness, with reports from various countries indicating an easy transition to digital technologies [25]. Throughout the pandemic, healthcare professionals became more receptive to the use of digital health solutions [26–28]. The results of this study demonstrate that a breakthrough has also occurred in Hungary regarding this matter. While there was no comprehensive survey specifically targeting PCPs on this topic in Hungary before, it is evident that digital tools have become an integral part of healthcare delivery [29].

The 33/2020 Decree of the Ministry of Human Resources (IX.16) on Telemedicine, Teleconsultations states that healthcare providers must possess the IT equipment needed for teleconsultations as well as detailed telemedicine use guidelines and patient information sheet, broadband, stable internet and virus protection. The decree also specifies what telemedicine interventions can be reimbursed. This has been in effect since the second wave of the pandemic. The technology and the regulatory framework are in place, so what is needed is the knowledge and the motivation to use it [30].

Our study assessed the digital health related experiences and attitudes of doctors during the third and fourth waves of the COVID pandemic, with a specific focus on PCPs. Summary of key findings is presented in the following table.

**Table:** 

**Summary of findings** ● **Knowledge**: On average, PCPs are familiar with fewer technologies, with a score of 8.53, compared to 9.21 for non-PCPs. PCPs are less familiar with international literature tracking online, augmented reality, virtual reality, 3D printing, artificial intelligence applications in healthcare, robotics, and nanotechnology. They are more familiar with telemedicine (remote consultations) and social media for patient communication, which facilitate easier communication with patients ● **Usage**: PCPs use telemedicine, social media for patient communication, and portable diagnostic devices significantly more frequently than non-PCPs ● **Patient demands**: PCPs reported more patient needs than non-PCPs: an average of 4.47 digital technology-related demands (email communication, sharing and discussing images and test results, teleconsultation, the ability for patients to track their health status on smartphone, home health sensor use, and the use of social media for communication with the doctor) from their patients vs. 3.66. These needs ● **Advantages**: more PCPs believe that the use of digital tools improves diagnostic capabilities, reduces the number of doctor-patient meetings, and makes healthcare delivery more efficient. In contrast, fewer PCPs than other physicians believe that the use of digital tools is convenient, saves time for the doctor, and reduces the likelihood of errors. The strongest impact on perceived benefits of digital health solutions is shown by the number of frequently or daily used technologies (Beta = 0.286), followed by the demand for knowledge related to digital device usage (0.223), and the perceived needs from patients, which also exert a positive effect on perceived benefits (Beta = 0.166). Based on the qualitative part of the research, the main benefits mentioned by the interviewed PCPs were: more efficient patient care, simpler way to inform patients and the easier way of consultation with specialist thus eliminating geographical inequalities ● **Disadvantages**: more PCPs believe that the use of digital tools frustrates patients, increases the administrative burden on doctors, poses extra costs to practices, and increases the risk of burnout. In contrast to non-PCPs, fewer PCPs think that it can lead to overdiagnosis, that patients misinterpret the shared health data, and that faulty technology jeopardises patients' recovery. Based on the interviews, depersonalisation, potential technical problems and the more complicated work-life balance were among the most important disadvantages of digital health ● **Prerequisites for further development**: More PCPs than non-PCPs indicated that they would need financial incentives, postgraduate training, other continuing education opportunities, and better cooperation and commitment from patients. In contrast, fewer PCPs considered the accessibility of technologies and evidence-based research to be necessary ● **Gender differences**: o Men use significantly more technology than women o Women have a significantly higher mean for knowledge needs than men o Men perceived more advantages while women reported more disadvantages of digital health solutions ● **Age differences:** o The number of known digital health tools decreases with age of PCPs o Younger PCPs use more devices than older PCPs o Younger PCPs see more advantages from digital health solutions than older PCPs o As age of PCPs increases, the perception of patient needs decreases o According to the interviews older doctors can be motivated by younger colleagues or even by their patients in the use of digital health technologies	

It can be observed that PCPs are much more open to technologies that facilitate patient communication and collaboration, while showing less interest in technologies supporting clinical work. In part this may stem from the nature of primary care medicine. Primary care in Hungary is not high technology-based. The essence of primary care is communication with the patient and decision whether higher level work-up and specialist referral is needed. Hence the digital health solutions used here are also those that serve communication and cooperation functions.

One of the key findings from our study underscores the influential role of patients' expectations and engagement in shaping their PCPs' attitudes towards digital technologies. As PCPs reported more patient demands for digital tools than non-PCPs, based on these findings, we can infer that PCPs are not only gatekeepers in the overall healthcare system, but they can also be considered potential digital gatekeepers. Moreover, their regular contact with patients positions them to potentially take on an important role in patient education regarding digital health tools. Although the study did not specifically address the question of patient education, it can be reasonably assumed that the digital transformation of healthcare may not be fully feasible without the active participation of PCPs in educating patients about these technologies.

Based on the findings of our research, it can be stated that the role of PCPs in patient communication and engagement is crucial particularly in using technologies that enhance communication like telemedicine. While AI technologies are expected to assist significantly, they are unlikely to replace the PCPs in this regard. Instead, they can greatly aid the work of PCPs.

According to the latest representative population survey 2024 of our research team, after the COVID-19 pandemic, patients' needs have increased: more than 80% of patients want to communicate with their doctor by email, 60% are open to telehealth, of those more than 80% would like to use a health sensor, nearly the same number would like their doctor to recommend trusted internet sources. A high proportion (over 70%) also indicate that they would like to receive recommendations for apps from their doctor [31].

Since the 2000s, the concept of e-patients has emerged, referring to individuals who actively participate in their healthcare by supporting digital technology. Empowerment, a key aspect of being an e-patient, involves gaining control over health-related decisions through understanding their role, acquiring knowledge, developing skills, and having a supportive environment. The COVID-19 pandemic has accelerated this trend, highlighting the essential role of digital tools in healthcare. This shift requires a cultural transformation in healthcare systems, fostering a new paradigm where patients and providers adapt to technological advancements for improved health outcomes [32].

Of the demographic variables (age, gender, type of settlement), age was found to have the most significant effect on digital health knowledge, use and intended use. One of the key prerequisites for the utilization of digital technologies is associated knowledge. This poses less of a challenge for the younger generation (both doctors and patients). However, considering that 55.9% of Hungarian PCPs are 60 years old or older it becomes particularly important in their case to provide adequate training, knowledge transfer, and potentially develop protocols. Peer support from junior colleagues is also essential in the digital transformation process. Since this is a rapidly evolving field, training should not be limited to older individuals alone. Continuous learning related to digital technologies is essential for younger generations alongside their constant professional development. The importance of training is also underscored by both the interviews and the multivariate analysis which reveal that individuals with more knowledge, expertise, and experience are more likely to recognise the benefits of digital solutions.

The results highlighted several key factors PCPs deemed necessary for the effective adoption of digital health technologies. Financial incentives were a primary concern, as many PCPs expressed that without proper funding and reimbursement models, integrating these technologies into their practices would be difficult. Additionally, they emphasized the need for improved accessibility to the technologies, particularly in rural areas, alongside clear ethical and legal regulations to ensure safe and standardized usage. Professional protocols and guidelines were also mentioned as crucial, as many PCPs felt that formal training and continuous education opportunities would be essential to confidently incorporate digital tools into their everyday practice.

The significance of patient demand and the mutual impact of openness between doctors and patients should also be emphasised, as they contribute to a reinforcing process. Based on previous experiences, respondents have a good perception of the advantages and disadvantages of digital solutions. The perceived benefits include increased healthcare capacity and improved efficiency of care. Among the disadvantages, PCPs are primarily concerned about potential patient frustration, increased administrative burdens and additional costs, and an increased likelihood of burnout. During the interviews, several participants also highlighted depersonalisation of doctor-patient relationships, blurred work-life boundaries, and potential negative consequences of technical issues.

While the degree of benefits perceived by PCPs is significantly influenced by age according to our initial assumptions and interview findings, this does not appear in the results of linear regression directly, because the use of digital technologies covers it up. Gender has a significant effect in the multivariate model as male PCPs perceive more advantages of digital healthcare solutions compared to female practitioners (this effect could not be seen in bivariate model above). However, a comparison of means shows that female PCPs use significantly more digital health tools on a regular basis. This also highlights the importance of training.

### Strengths and limitations

During the COVID-19 pandemic, digital tools quickly became an integral part of healthcare, and their availability was limited to online platforms at certain stages of the epidemic, particularly in primary care. This means that respondents were able to provide authentic opinions based on their everyday experiences. However, it is important to acknowledge that the crisis situation and the necessity of transitioning to digital platforms also presented significant challenges for the interviewed doctors. This duality should be considered when interpreting the responses.

The survey was conducted at the time of the COVID-19 epidemic, and since then, as the epidemic has subsided, the demand for and attitudes towards digital health solutions have naturally changed. This is certainly worth bearing in mind when interpreting the results. In the near future, it will be necessary to assess the post-COVID digital healthcare landscape, as it will reveal the true impact of the pandemic and what remains after its effects subside.

A further limitation of the survey is that the age distribution of respondents is slightly younger than the age distribution of primary care doctors in Hungary.

## Conclusions

Based on the results of this study, our research questions can be answered as follows:

- *What are the digital health-related attitudes and experiences of Hungarian PCPs?* – Hungarian primary care physicians are generally less familiar with advanced digital technologies (e.g., AI, AR/VR, robotics), but they are more familiar with telemedicine and patient communication tools such as social media. They use telemedicine and portable diagnostic devices more frequently and report more patient demands for digital technologies compared to non-primary care physicians.
- *Are there any differences between the digital health-related attitudes and experiences of PCPs and non-primary care medical doctors in Hungary and what are they?* – Yes, there are differences. PCPs are less familiar with advanced technologies but make more frequent use of telemedicine and social media for patient communication. They experience more patient demands related to digital health and are more likely to believe that digital tools improve diagnostic capabilities and healthcare efficiency. However, PCPs perceive fewer conveniences and more challenges, such as increased administrative burden and burnout, compared to non-PCPs.
- *Are there any effects of gender, age and settlement type on digital health-related attitudes and experiences of Hungarian PCPs?* – Yes, gender and age have significant effects. Male PCPs use more technologies, while female PCPs express a greater need for knowledge. Men perceive more benefits from digital tools, while women report more disadvantages. As PCPs age, the number of digital tools they are familiar with decreases, and they perceive fewer patient needs. Younger PCPs use more digital devices and view digital health solutions more favorably, while older doctors may be motivated by younger colleagues or patients to adopt digital health technologies.

Our study highlights the critical role of primary care physicians in the adoption and integration of digital health technologies, which was especially pronounced during the COVID-19 pandemic. The findings indicate that while PCPs in Hungary have become more open to technologies that facilitate communication and collaboration with patients, there remains qualm towards tools that support clinical work. This selective adoption is reflective of the nature of primary care, which prioritises patient interaction and decision-making over high-technology interventions. Thus, the digital transformation in healthcare largely depends on the involvement of PCPs, who serve as both gatekeepers and educators in this evolving landscape.

Moreover, patient expectations and engagement have become major drivers of this digital shift. The pandemic has not only accelerated the acceptance of digital tools but also highlighted the growing demand from patients for digital communication and health management solutions. The concept of the empowered 'e-patient' is gaining ground, requiring a cultural change in healthcare that supports technological advances. For this transformation, continuous education and training are essential, especially considering the aging PCP workforce in Hungary. The emphasis on both patient demand and PCP readiness highlights the need for a unified approach to digital health, ensuring that both patients and providers are prepared to navigate and benefit from these advancements.

It is crucial to draw attention to the fact that health policymakers need to actively support the wider adoption of digital tools, whether through knowledge dissemination, protocol development, or infrastructure enhancement.

## Supplementary Information

Supplementary Material 1.

Supplementary Material 2.

## Data Availability

The datasets used and/or analysed during the current study are available from the corresponding author on reasonable request.

## Abbreviations

DESI: Digital Economy and Society Index
EESZT: Hungarian Electronic Health Record System
HER: Electronic Health Records
EMR: Electronic Medical Record
GP: General Practitioner
HMC: Hungarian Medical Chamber
PCP: Primary Care Physician
