# Supplementary material for: Exploring the attitudes and experiences of Hungarian primary care physicians on the utilisation of digital health solutions
Source: BMC Prim Care. 2024 Nov 14;25:396. doi: 10.1186/s12875-024-02642-8 (PMC11566197; doi:10.1186/s12875-024-02642-8)
Supplement: Supplementary file 1 — Supplementary Material 1. [file 12875_2024_2642_MOESM1_ESM.pdf]

# Digital Health - Medical Survey

## 1. Greetings

Dear Colleague!

On behalf of the "Digital Health" working group of the Institute of Behavioural Sciences of Semmelweis University we would like to ask for your help (https://semmelweis.hu/digitalhealth/). Within the project "E-doctors and e-patients in Hungary: the role and opportunities of digitalisation in healthcare", 2020 NRDI-1 grant (FK 134372), we wish to examine the impact of digitalisation in healthcare from both a medical and a patient perspective. We would be grateful if you could take some time to fill in our questionnaire. Thank you!

Research ethics approval number: IV/10927/2020/ECU

Dr. Zsuzsa Győrffy and Dr. Edmond Girasek

## 2. Socio-Demographic Questions

2.1 In which year were you born? --------------

2.2 Your gender: ☐ male ☐ female

2.3 In which country are you working presently? ☐ Hungary ☐ abroad

2.4 IF WORKING IN HUNGARY: Which county do you currently work in (If it is in more than one county indicate the county where you work primarily!)

| | | |
|---|---|---|
| ☐ Budapest | ☐ Baranya | ☐ Borsod-Abaúj-Zemplén |
| ☐ Bács-Kiskun | ☐ Békés | ☐ Csongrád-Csanád |
| ☐ Fejér | ☐ Győr-Moson-Sopron | ☐ Hajdú-Bihar |
| ☐ Heves | ☐ Jász-Nagykun-Szolnok | ☐ Komárom-Esztergom |
| ☐ Nógrád | ☐ Pest | ☐ Somogy |
| ☐ Szabolcs-Szatmár-Bereg | ☐ Tolna | ☐ Vas |
| ☐ Veszprém | ☐ Zala | |

2.5 IF WORKING ABROAD: in which country are you currently working?

---------------------------------------------------------------

2.6 Which faculty did you get your degree from? ☐ General Medicine ☐ Dentistry

## 3. Questions related to medical work

3.1 Among the following, where would you mostly categorize your work?

☐ Currently, I work in patient care.

☐ Currently, I do not work in patient care but in healthcare/health research.

☐ My current job is not related to healthcare.

3.2 Please select the statement that best applies to you:

- ☐ resident/medical intern
- ☐ doctor/dentist working outside of specialization training
- ☐ medical specialist
- ☐ research physician
- ☐ retired doctor
- ☐ substitute doctor
- ☐ entrepreneur running your own private practice
- ☐ university lecturer/professor
- ☐ university student/researcher (e.g. PhD, university, etc.)
- ☐ on parental leave

3.3 Among the following options, please select the type that best represents your current primary workplace/occupation:

- ☐ university, college
- ☐ research institute
- ☐ clinic, national institute
- ☐ hospital
- ☐ outpatient clinic, other specialist institution
- ☐ primary care/primary healthcare institution
- ☐ private healthcare institution
- ☐ multinational corporation
- ☐ Hungarian enterprise/company (for individuals working abroad: national enterprise/company)
- ☐ government administration, administration
- ☐ healthcare and related professional chambers, associations, societies, organizations
- ☐ other

3.4 If you chose the "other" category in the previous question, please specify:

------------------------------------------------------------------------

3.5 Among your workplaces other than your main job, do you have any in the private sector (private clinic, company, etc.)?

- ☐ Yes
- ☐ No
- ☐ I don't have any workplaces other than my main job.

3.6 In which city is the location of your main job?

- ☐ Capital city
- ☐ County seat
- ☐ City
- ☐ Village, township, rural area

3.7 How many specialized certifications do you have?

- ☐ I don't have any specialized certifications.
- ☐ 1
- ☐ 2
- ☐ 3
- ☐ More than 3

3.8 In what field do you work?

- ☐ Anesthesiology and intensive therapy
- ☐ Maxillofacial surgery
- ☐ Internal medicine
- ☐ Dermatology
- ☐ Family medicine
- ☐ Pediatrics
- ☐ Dentoalveolar surgery
- ☐ Physical medicine and rehabilitation
- ☐ Occupational medicine
- ☐ Orthodontics
- ☐ Otorhinolaryngology
- ☐ Gastroenterology
- ☐ Geriatrics
- ☐ Child and adolescent psychiatry
- ☐ Pediatric dentistry
- ☐ Pediatric surgery
- ☐ Military medicine, disaster medicine
- ☐ Neurosurgery
- ☐ Forensic medicine
- ☐ Infectious diseases
- ☐ Cardiology
- ☐ Conservative dentistry and prosthodontics
- ☐ Public health, epidemiology
- ☐ Preventive medicine and public health
- ☐ Neurology
- ☐ Nuclear medicine
- ☐ Orthopedics and traumatology
- ☐ Medical laboratory diagnostics
- ☐ Medical microbiology
- ☐ Emergency medicine and urgent care
- ☐ Periodontology
- ☐ Pathology
- ☐ Psychiatry
- ☐ Radiology
- ☐ Aviation medicine
- ☐ Rheumatology
- ☐ Surgery
- ☐ Ophthalmology
- ☐ Cardiac surgery
- ☐ Obstetrics and gynecology
- ☐ Transfusion medicine
- ☐ Pulmonology
- ☐ Urology
- ☐ Other

If you chose "Other," please explain your field of work: ------------------------------------------------

3.10 After obtaining your medical degree, have you worked as a doctor/dentist abroad for a shorter or longer period of time? *Please take into account any current foreign employment as well.*

- ☐ Yes, I have.
- ☐ Yes, I am currently working abroad.
- ☐ No, I haven't.

3.11 In which country? ------------------------------------------------

3.12 How often do you use the internet for your work?

| | | |
|---|---|---|
| ☐ Daily | ☐ Multiple times a day | ☐ Several times a week |
| ☐ Once a week | ☐ Once a month | ☐ Never |

| | No, never | Yes, occasionally | Yes, frequently | Yes, very often |
|---|---|---|---|---|
| 3.13 Do you recommend websites to your patients? | ☐ | ☐ | ☐ | ☐ |
| 3.14 Do you recommend mobile applications to your patients? | ☐ | ☐ | ☐ | ☐ |
| 3.15 Do you recommend social media sources/groups to your patients? | ☐ | ☐ | ☐ | ☐ |

| | | | | | | | |
|---|---|---|---|---|---|---|---|
| 3.16 What do you think about your patients' use of the internet for health-related purposes? | I completely oppose it | ☐ | ☐ | ☐ | ☐ | ☐ | I fully support it |
| 3.17 Compared to your patients, how would you rate your own internet usage knowledge on the following five-point scale? | I struggle much worse in the online world than my patients. | ☐ | ☐ | ☐ | ☐ | ☐ | I excel much better in the online world than my patients. |

## 4. Digital Technologies

Below, we will ask you questions regarding digital technologies to inquire whether you are familiar with them and to what extent you use each technology.

**Participating in online conferences and trainings**

4.1 Are you familiar with this technology - Participating in online conferences and trainings?

☐ I am not familiar with it. ☐ I am familiar with it.

| | | | | | | |
|---|---|---|---|---|---|---|
| 4.2 How often do you use it? | I don't use it at all. | ☐ | ☐ | ☐ | ☐ | ☐ | I use it intensively (e.g., every day) |
| 4.3 How willing are you to use it in the next 3 years? | Not willing at all. | ☐ | ☐ | ☐ | ☐ | ☐ | Completely willing. |

**Tracking international literature, trends, and data online**

4.4 Are you familiar with this technology - Tracking international literature, trends, and data online?

☐ I am not familiar with it. ☐ I am familiar with it.

| | | | | | | | |
|---|---|---|---|---|---|---|---|
| 4.5 How often do you use it? | I don't use it at all. | ☐ | ☐ | ☐ | ☐ | ☐ | I use it intensively (e.g., every day) |
| 4.6 How willing are you to use it in the next 3 years? | Not willing at all. | ☐ | ☐ | ☐ | ☐ | ☐ | Completely willing. |

**Telemedicine, remote visit**

4.7 Are you familiar with this technology - Telemedicine, remote visit?

☐ I am not familiar with it. ☐ I am familiar with it.

| | | | | | | | |
|---|---|---|---|---|---|---|---|
| 4.8 How often do you use it? | I don't use it at all. | ☐ | ☐ | ☐ | ☐ | ☐ | I use it intensively (e.g., every day) |
| 4.9 How willing are you to use it in the next 3 years? | Not willing at all. | ☐ | ☐ | ☐ | ☐ | ☐ | Completely willing. |

**Smartphone applications, apps**

4.10 Are you familiar with this technology - Smartphone applications, apps?

☐ I am not familiar with it. ☐ I am familiar with it.

| | | | | | | | |
|---|---|---|---|---|---|---|---|
| 4.11 How often do you use it? | I don't use it at all. | ☐ | ☐ | ☐ | ☐ | ☐ | I use it intensively (e.g., every day) |
| 4.12 How willing are you to use it in the next 3 years? | Not willing at all. | ☐ | ☐ | ☐ | ☐ | ☐ | Completely willing. |

**Healthcare-related social media, communication with patients, information sharing**

4.13 Are you familiar with this technology - Healthcare-related social media, communication with patients, information sharing?

☐ I am not familiar with it. ☐ I am familiar with it.

4.14 How often do you use it?

I don't use it at all. ☐ ☐ ☐ ☐ ☐ I use it intensively (e.g., every day)

4.15 How willing are you to use it in the next 3 years?

Not willing at all. ☐ ☐ ☐ ☐ ☐ Completely willing.

**Home-usable healthcare sensors, smart devices**

4.16 Are you familiar with this technology - Home-usable healthcare sensors, smart devices?

☐ I am not familiar with it. ☐ I am familiar with it.

4.17 How often do you use it?

I don't use it at all. ☐ ☐ ☐ ☐ ☐ I use it intensively (e.g., every day)

4.18 How willing are you to use it in the next 3 years?

Not willing at all. ☐ ☐ ☐ ☐ ☐ Completely willing.

**Portable diagnostic devices (e.g., ultrasound, mobile ECG)**

4.19 Are you familiar with this technology - Portable diagnostic devices (e.g., ultrasound, mobile ECG)?

☐ I am not familiar with it. ☐ I am familiar with it.

4.20 How often do you use it?

I don't use it at all. ☐ ☐ ☐ ☐ ☐ I use it intensively (e.g., every day)

4.21 How willing are you to use it in the next 3 years?

Not willing at all. ☐ ☐ ☐ ☐ ☐ Completely willing.

**Augmented reality (e.g., surgical practice)**

4.22 Are you familiar with this technology - Augmented reality (e.g., surgical practice)?

☐ I am not familiar with it. ☐ I am familiar with it.

4.23 How often do you use it?

I don't use it at all. ☐ ☐ ☐ ☐ ☐ I use it intensively (e.g., every day)

4.24 How willing are you to use it in the next 3 years?

Not willing at all. ☐ ☐ ☐ ☐ ☐ Completely willing.

**Use of Virtual Reality (e.g., pain management, psychotherapy)**

4.25 Are you familiar with this technology - Use of Virtual Reality (e.g., pain management, psychotherapy)?

☐ I am not familiar with it. ☐ I am familiar with it.

4.26 How often do you use it?

I don't use it at all. ☐ ☐ ☐ ☐ ☐ I use it intensively (e.g., every day)

4.27 How willing are you to use it in the next 3 years?

Not willing at all. ☐ ☐ ☐ ☐ ☐ Completely willing.

**3D printing (e.g., dental, surgical solutions)**

4.28 Are you familiar with this technology - 3D printing (e.g., dental, surgical solutions)?

☐ I am not familiar with it. ☐ I am familiar with it.

4.29 How often do you use it?

I don't use it at all. ☐ ☐ ☐ ☐ ☐ I use it intensively (e.g., every day)

4.30 How willing are you to use it in the next 3 years?

Not willing at all. ☐ ☐ ☐ ☐ ☐ Completely willing.

**Artificial intelligence solutions in medical decision-making (radiology, pathology, ophthalmology, diagnostic solutions)**

4.31 Are you familiar with this technology - Artificial intelligence solutions in medical decision-making (radiology, pathology, ophthalmology, diagnostic solutions)?

☐ I am not familiar with it. ☐ I am familiar with it.

4.32 How often do you use it?

I don't use it at all. ☐ ☐ ☐ ☐ ☐ I use it intensively (e.g., every day)

4.33 How willing are you to use it in the next 3 years?

Not willing at all. ☐ ☐ ☐ ☐ ☐ Completely willing.

**Robotics (e.g., surgical robots, disinfection robots, delivery robots)**

4.34 Are you familiar with this technology - Robotics (e.g., surgical robots, disinfection robots, delivery robots)?

☐ I am not familiar with it. ☐ I am familiar with it.

4.35 How often do you use it?

I don't use it at all. ☐ ☐ ☐ ☐ ☐ I use it intensively (e.g., every day)

4.36 How willing are you to use it in the next 3 years?

Not willing at all. ☐ ☐ ☐ ☐ ☐ Completely willing.

**Nanotechnology (e.g., ingestible diagnostic devices)**

4.37 Are you familiar with this technology - Nanotechnology (e.g., ingestible diagnostic devices)?

☐ I am not familiar with it. ☐ I am familiar with it.

4.38 How often do you use it?

I don't use it at all. ☐ ☐ ☐ ☐ ☐ I use it intensively (e.g., every day)

4.39 How willing are you to use it in the next 3 years?

Not willing at all. ☐ ☐ ☐ ☐ ☐ Completely willing.

## 5. Digital Health

5.1 Do you experience demand from your patients regarding the following? *You can select any number of answers.*

- ☐ Email communication for scheduling appointments online.
- ☐ Teleconsultation (via Skype or video chat).
- ☐ Using home health sensors.
- ☐ Using social media for communication with you.
- ☐ Sharing and discussing images and test results.
- ☐ Monitoring changes in their health status through their smartphones.
- ☐ Recommending websites with valid medical information.

5.2 What do you think it would take for you to use digital health solutions in your practice? *You can select any number of answers.*

- ☐ Financial incentives (e.g., support for acquiring certain tools).
- ☐ Postgraduate training.
- ☐ Availability and accessibility of technologies.
- ☐ Evidence-based research.
- ☐ Professional protocols.
- ☐ Dedicated time within working hours.
- ☐ Accessible professional materials (documents, online training, etc.).
- ☐ Other training opportunities.
- ☐ Recommendations from colleagues.
- ☐ Ethical and legal regulations.
- ☐ Data security protocols.
- ☐ Patient commitment and increased collaboration.

5.3 What do you think are the potential benefits of digital health solutions? *You can select any number of answers.*

- ☐ Improved efficiency.
- ☐ Improved diagnostic capabilities.
- ☐ Increased patient adherence and collaboration.
- ☐ Reduced the number of in-person doctor-patient meetings.
- ☐ Save time for the patient.
- ☐ Make your work more efficient.
- ☐ Improve the quality of care.
- ☐ Generate additional income for doctors.
- ☐ Improve doctor-patient communication
- ☐ Enhanced safety.
- ☐ Reduced burnout.
- ☐ Convenient.
- ☐ Save time for the doctor.
- ☐ Enable faster access to healthcare.
- ☐ Engage patients more actively in their own healing process.
- ☐ Reduce the likelihood of errors.
- ☐ Increase patient satisfaction.

5.4 What do you think could be the potential negative consequences of digital health solutions?
*You can select any number of answers.*

- ☐ Decreased quality of care.
- ☐ Potential for overdiagnosis.
- ☐ Increased possibility of misunderstandings in doctor-patient communication.
- ☐ Compromised confidentiality of patient data.
- ☐ Additional costs for practices.
- ☐ Increased likelihood of burnout.
- ☐ Frustration among patients.
- ☐ Misinterpretation of shared health data by patients.
- ☐ Faulty technology jeopardizing patient recovery.
- ☐ Increased administrative burdens on doctors.
- ☐ Limited patient proficiency in using digital technologies, placing a burden on the treating physician.

5.5 When you think about the use of digital health technologies, how do you feel?

- ☐ I don't know
- ☐ it doesn't trigger anything in me
- ☐ it scares me
- ☐ it evokes positive feelings
- ☐ I'm worried
- ☐ I find it exciting

5.6 To what extent do you perceive that doctors/healthcare workers in your environment are open to technological development and digitalization?

They are not open at all, they consider it burdensome. ☐ ☐ ☐ ☐ ☐ They are totally open-minded and eagerly anticipate every technological innovation.

5.7. What is your opinion on patient self-education (through online portals or potentially through smart devices in the future)?

- ☐ I consider it harmful and hindering, as it complicates the work of doctors (with more disadvantages than advantages)
- ☐ With careful usage, it can be particularly beneficial (with more advantages than disadvantages)
- ☐ I find it highly useful as it greatly assists the doctor and speeds up the work.
- ☐ In the future, with the increasing prevalence of smart devices, it could become very useful, although currently there are more disadvantages than advantages.
- ☐ I can't decide

5.8. Would you consider it important that knowledge about online healthcare is included in the medical curriculum?

Not at all ☐ ☐ ☐ ☐ ☐ Yes, completely

5.9. If you have any comments or suggestions, either on the questionnaire or on the topic, please feel free to share them:

| |
|---|
| |

Thank you for responding!
